# Heparin Forms Polymers with Cell-free DNA Which Elongate Under Shear in Flowing Blood

**DOI:** 10.1038/s41598-019-54818-3

**Published:** 2019-12-04

**Authors:** Joost C. de Vries, Arjan D. Barendrecht, Chantal C. Clark, Rolf T. Urbanus, Peter Boross, Steven de Maat, Coen Maas

**Affiliations:** 1 Department of Clinical Chemistry & Haematology, University Medical Center Utrecht, Utrecht University, Utrecht, The Netherlands; 2 Immunotherapy Laboratory, Laboratory for Translational Immunology, University Medical Center Utrecht, Utrecht University, Utrecht, The Netherlands

**Keywords:** Platelets, Cellular imaging, Cell biology, Fluorescence imaging, Thrombosis

## Abstract

Heparin is a widely used anticoagulant which inhibits factor Xa and thrombin through potentiation of antithrombin. We recently identified that the nucleic acid stain SYTOX reacts with platelet polyphosphate due to molecular similarities, some of which are shared by heparin. We attempted to study heparin in flowing blood by live-cell fluorescence microscopy, using SYTOX for heparin visualisation. Immunostaining was performed with monoclonal antibodies directed against various heparin-binding proteins. In addition, we studied modulation of heparin activity in coagulation assays, as well its effects on fibrin formation under flow in recalcified whole blood. We found that SYTOX-positive polymers appear in heparinised blood under flow. These polymers typically associate with platelet aggregates and their length (reversibly) increases with shear rate. Immunostaining revealed that of the heparin-binding proteins assessed, they only contain histones. In coagulation assays and flow studies on fibrin formation, we found that addition of exogenous histones reverses the anticoagulant effects of heparin. Furthermore, the polymers do not appear in the presence of DNase I, heparinase I/III, or the heparin antidote protamine. These findings suggest that heparin forms polymeric complexes with cell-free DNA in whole blood through a currently unidentified mechanism.

## Introduction

Heparin is a highly sulfated anionic polysaccharide of ±15 kDa, best known for its anticoagulant properties. It operates through potentiation of antithrombin (AT), which in turn inhibits factor Xa and thrombin^1,2^. In addition, heparin can bind several other proteins, primarily due to its high negative charge density. Among others, specific binding to P- and L-selectin has been reported^3,4^. Histones are another class of binding partners of interest. This interaction inhibits both the cytotoxic and proinflammatory effects of histones, as well as the anticoagulant effect of heparin^5–10^. In addition, the clinically applied antidote for heparin, protamine, is a histone-like DNA-binding protein which replaces histones in spermatogenesis to allow for DNA condensation^11^. Moreover, protamine is evolutionary linked to histones as it originates from the linker histone H1^12–14^.

Heparin can also interact with various platelet-bound and plasma-derived proteins, such as fibronectin^15^ and thrombospondin-1^16^. The interaction between platelet factor 4 (PF4)^17^ and heparin is well-known for its involvement in heparin-induced thrombocytopenia (HIT)^18^ - a relatively rare but serious side-effect of heparin treatment. Interestingly, it was recently shown that fibronectin protects against HIT through disruption of the heparin-PF4 complex^15^. For other heparin-protein interactions, the effects on heparin function are less clear.

We recently found that SYTOX, which is a commonly-used staining reagent for detection of extracellular DNA, also binds to platelet polyphosphate^19^. This interaction is likely to be based on molecular similarities between polyphosphate and DNA, such as composition (DNA has a sugar-phosphate backbone) and a net negative charge. As heparin is a highly anionic polysaccharide, we considered the possibility of SYTOX also binding to heparin based on molecular similarities to DNA as well. We performed live-cell imaging of platelet aggregation under flow in heparinised human whole blood and attempted to visualise heparin using SYTOX to experimentally address this hypothesis. During these experiments, we made a series of remarkable observations.

## Results

### Polymers emerge in heparinised whole blood under flow that can be visualised with SYTOX

**Figure 1:**
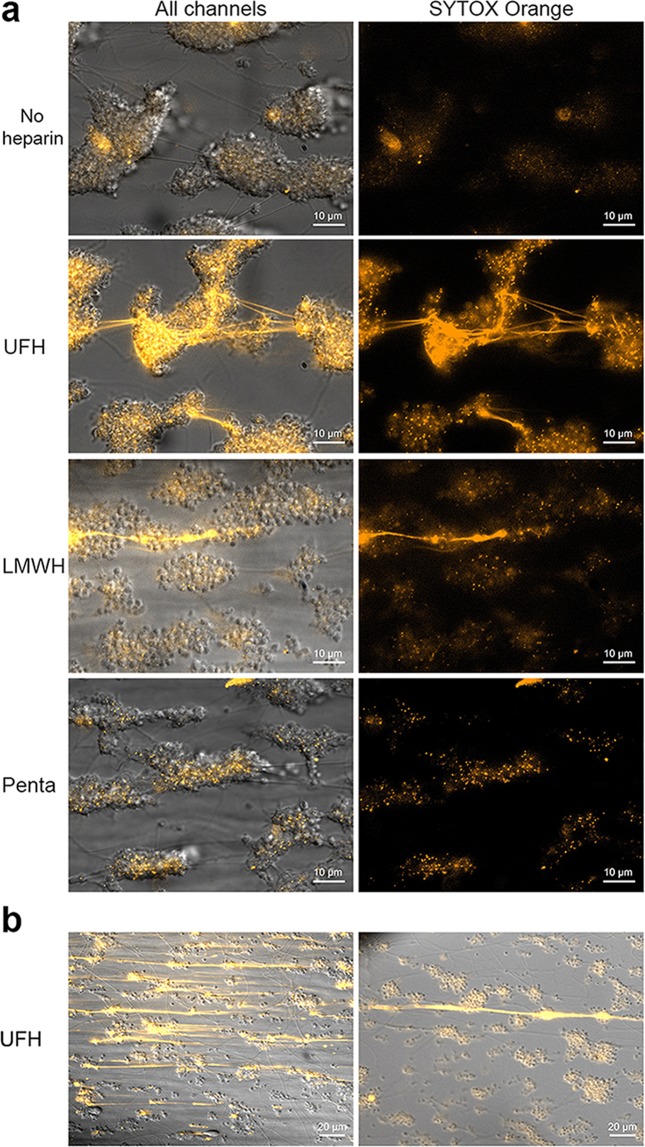
Polymers emerge in heparinised whole blood under flow that can be visualised with SYTOX. (**a**) SYTOX-positive polymers are observed in the presence of UFH or LMWH (10 IU/mL), but not in the absence of either, nor in the presence of pentasaccharide (penta). (**b**) Experimental variation in the number (left panel) and length (right panel) of the polymers. Experiments for all conditions were performed at least thrice. The scale bars represent 10 µm in panel (a) and 20 µm in panel (b).

**Figure 2:**
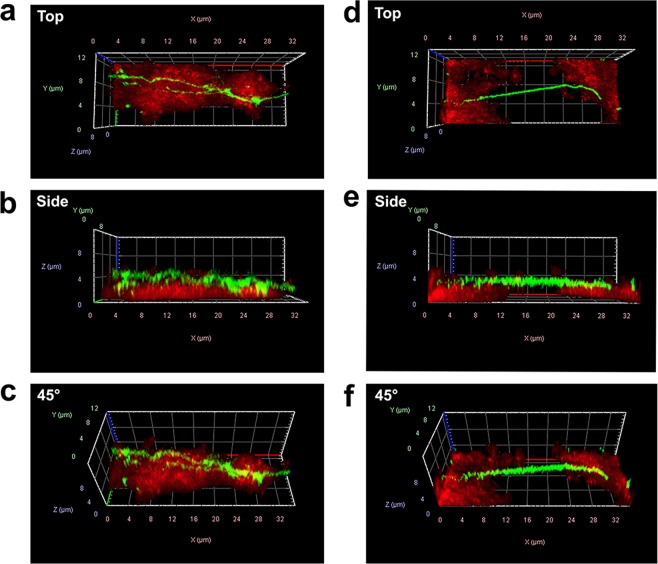
Polymers associate with the platelet aggregate surface. Three-dimensional analyses of platelet aggregates (stained through the platelet-specific receptor GPIb-α in red) and SYTOX Orange (false-coloured in green with ZEN software to enhance contrast). (**a**–**c**) Polymers accumulate on top of the platelet aggregate surface (**d**–**f**). Polymers that bridge multiple aggregates do not associate with the collagen-coated surface.

When citrated whole blood is supplemented with heparin (unfractionated heparin; UFH or low molecular weight heparin; LMWH) and perfused over immobilised collagen, SYTOX-positive polymers are observed (Fig. 1a). These polymers are invisible in the corresponding DIC images. Furthermore, they are not seen in the absence of heparin or in the presence of pentasaccharide (fondaparinux; quantification in Fig. S1), leading us to hypothesise that heparin is incorporated into these polymers. In a two-stage experiment, we found that SYTOX itself is not involved in the polymerization process (e.g. through cation-anion interactions). First, we allowed platelet aggregates to form in heparinised whole blood (i.e. without SYTOX). Subsequently, we perfused SYTOX in buffer (now in the absence of heparin) over the cover slides, revealing SYTOX-positive polymers (Fig. S2). Typically, these polymers appear to adhere to the platelet aggregate surface. Three-dimensional imaging of platelet aggregates (platelets visualised through the platelet-specific marker glycoprotein Ib-α; GPIb-α) shows that polymers are located on top of platelet aggregates (Fig. 2). In cases where polymers bridge multiple aggregates, they do not appear to associate with the collagen surface. There is inter-subject variation regarding the number of polymers that form; some form very high numbers (example Fig. 1b; left panel). In addition, there is variation in polymer length (within experiments), with some polymers being very large (larger than field of view of 225 µm; example Fig. 1b right panel).

### Polymer length is a function of shear rate

**Figure 3:**
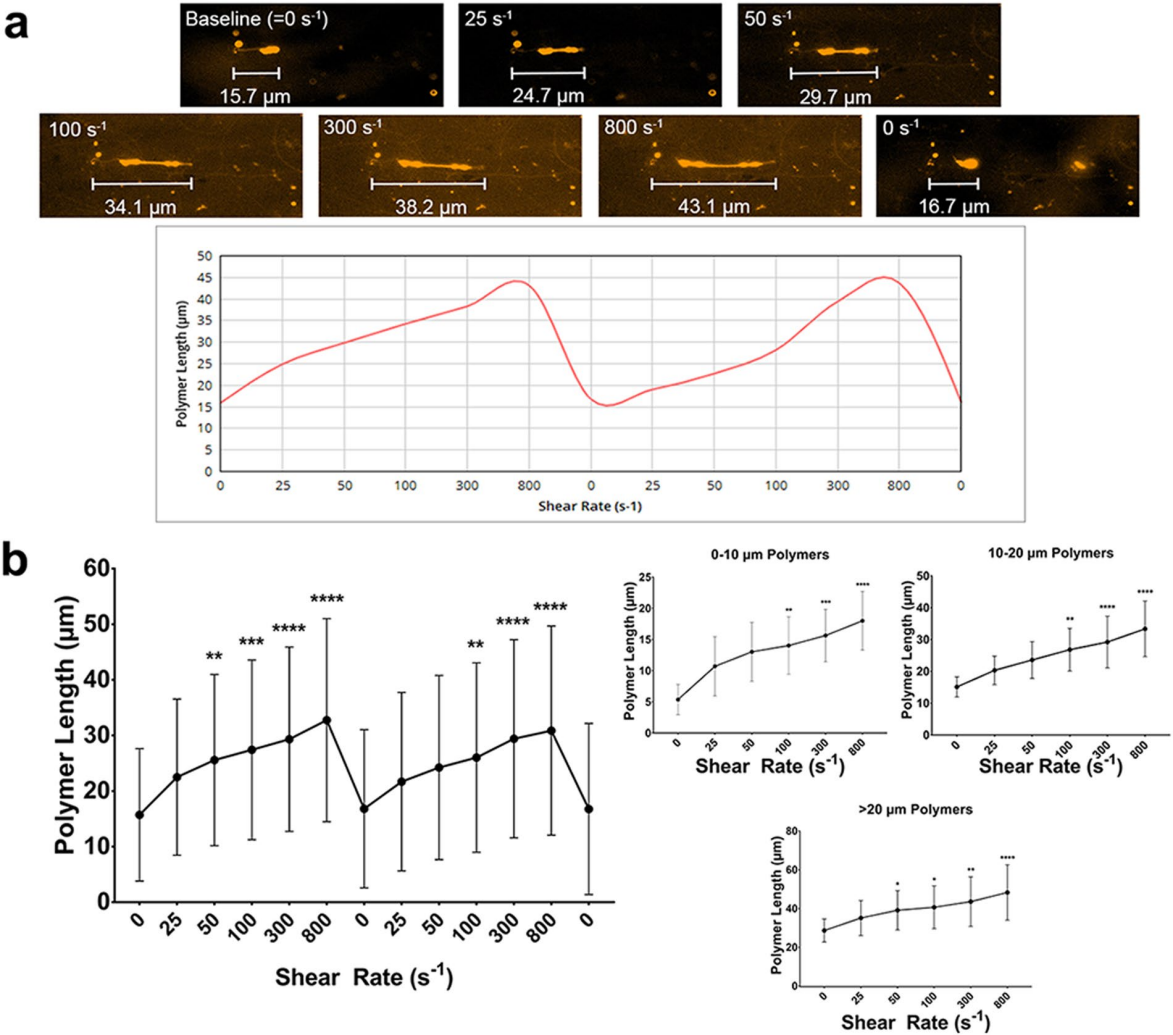
SYTOX-positive polymer length is a function of shear rate. (**a**) Representative images of a polymer, exposed to two cycles of shear rate variation. Polymer length of this example is plotted in the graph below. (**b**) Compound data from shear dependency experiments, analyzed for two cycles (n = 12 polymers). Right panels: polymer elongation data from polymers (single cycles were analyzed), stratified according to baseline length of the polymers. Sample sizes are n = 8 (0–10 µm), n = 10 (10–20 µm), and n = 7 (>20 µm). Data represent mean +/− SD and were analyzed using a Friedman test with Dunn correction, *p > 0.05, **p > 0.01, ***p > 0.001, ****p > 0.0001 compared to baseline (0 s^−1^).

During our experiments, we observed that polymer length was variable. To further examine this phenomenon, we performed a series of experiments where polymer length was measured in cycles of increasing shear rates. Representative behavior of a polymer shown in Supplemental Video 1 and Fig. 3a. These polymers elongate with increasing shear rates, and are significantly longer than baseline lengths from shear rates of 50 s^−1^ and higher (Fig. 3b; *p* < 0.05). There is no major difference in the degree of elongation between polymers with different baseline lengths, as displayed by the stratified data. Interestingly, polymers return to (near) baseline lengths in the absence of shear (0 s^−1^) between and after the two cycles, indicating that polymer elongation is reversible.

### SYTOX-positive polymers contain histones, but not antithrombin, Platelet Factor 4 or Fibronectin

**Figure 4:**
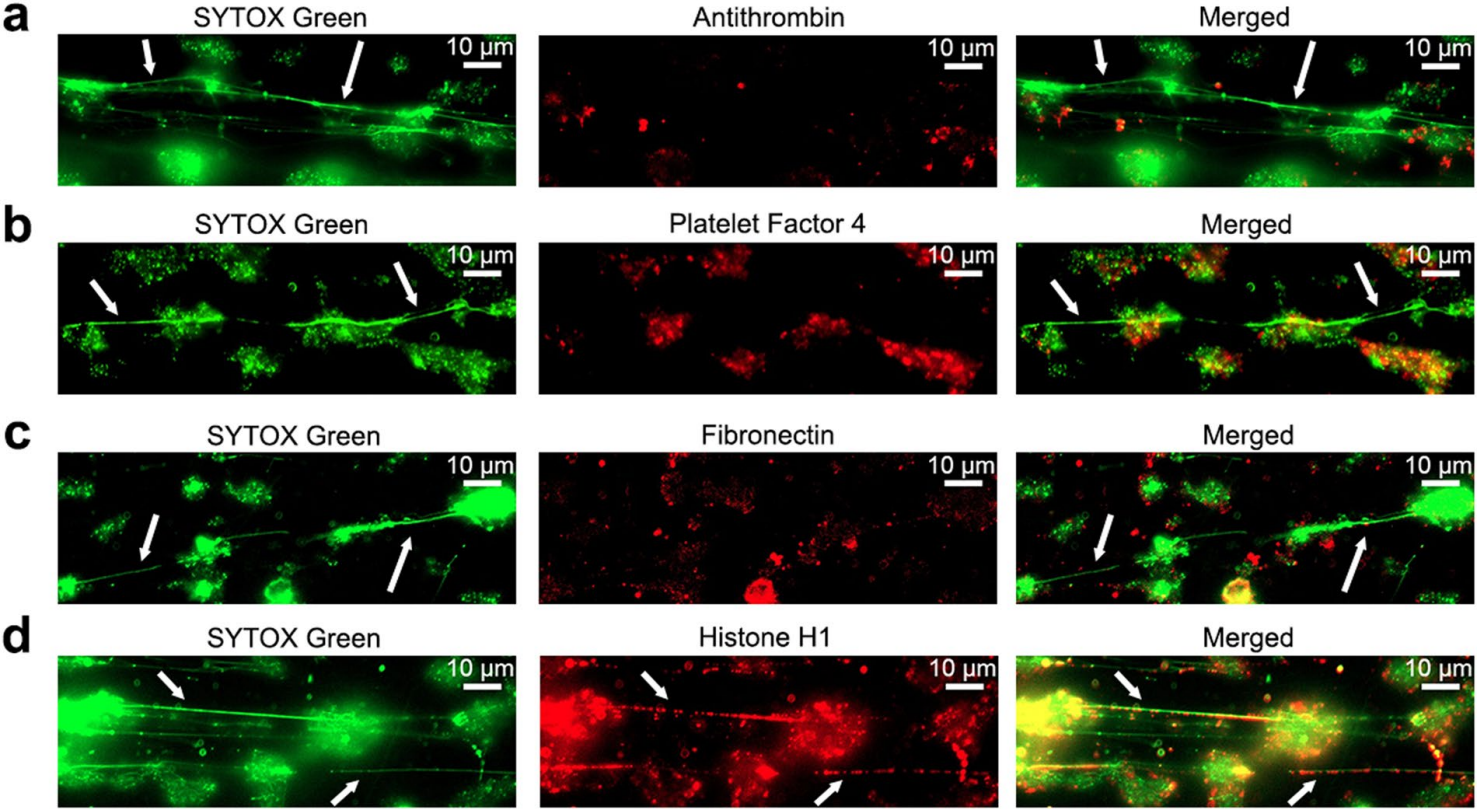
SYTOX-positive polymers contain histones, but not antithrombin, Platelet Factor 4 or Fibronectin. (**a**) Co-localization between SYTOX Green and antithrombin. (**b**) Platelet Factor 4. (**c**) Fibronectin, and (**d**) histones by immunostaining fixed coverslides obtained from citrated whole-blood flow experiments over immobilised collagen surfaces. Images are representative for at least three separate experiments. In all figures, the scale bars represent  10 µm; examples of SYTOX-positive polymers are indicated by white arrows.

We next investigated whether polymers contain protein binding partners of heparin by immunostaining. Our first candidate was AT. Surprisingly, using different anti-AT antibodies (3 monoclonal antibodies, 1 polyclonal antibody), we failed to show the presence of AT on the polymers in samples that were fixed after perfusion experiments (Fig. 4a). In a similar manner, we failed to identify PF4^17,18^ or fibronectin^15^ on the polymers (Fig. 4b,c). Furthermore, VWF^20^ showed no colocalization with the SYTOX-positive polymers (Fig. S3). In contrast, we found that these polymers are strikingly positive for histones^5–10^, which can be seen both directly under flow and by immunohistochemistry in fixed samples (Fig. 4d). For these experiments, we used an antibody that reacts with histone H1, histone H3 (and to a lesser extent H2B). No signal was observed in control experiments with isotype control antibodies or experiments in which the primary antibody was omitted (Fig. S4). We next aimed to pinpoint the source of the histones. We therefore investigated heparin preparations and citrated human plasma for the presence of histones. Coomassie staining of the heparin preparations showed that these were completely devoid of protein (data not shown), ruling out the possibility of histones being present in these preparations. Thus, we considered the citrated blood the most likely source of histones, which was corroborated by a report showing histones to be present in human whole blood from healthy controls in a concentration of 720 µg/L (range 451–989 µg/L), and 180 µg/L (range 8–352 µg/L) for H2B and H3 respectively^21^.

### Histones reverse the anticoagulant effects of heparin

**Figure 5:**
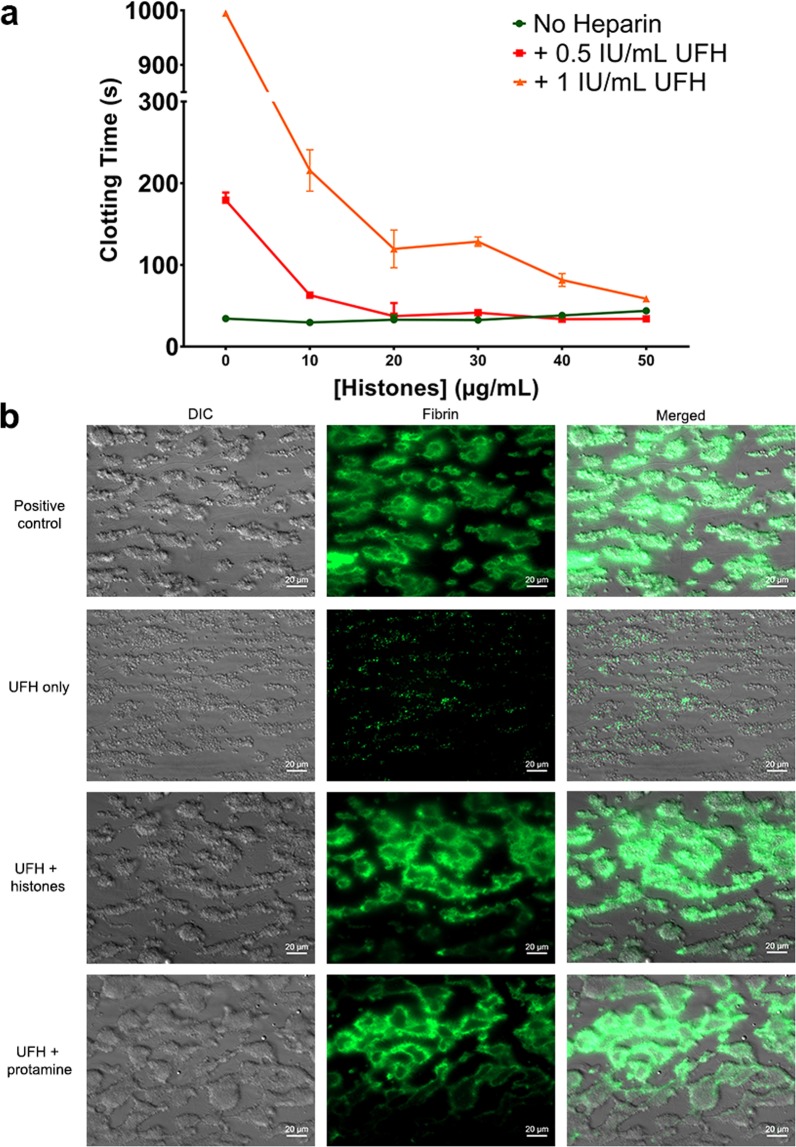
Histones reverse the anticoagulant effects of heparin. (**a**) Citrated plasma was supplemented with 0.5 or 1 IU UFH, as well as a concentration range of exogenous histones. Clotting times (aPTT) experiments were subsequently determined in triplicate. Data represent means +/− SD. (**b**) Fibrin formation (green) in recalcified citrated whole blood on immobilised collagen under flow. Supplementation with 10 IU/mL UFH completely inhibits fibrin formation. This is reversed by either 500 µg/mL histones or 125 µg/mL protamine. Representative images were taken at the onset of fibrin formation (i.e. before the flow chamber becomes obstructed), in conditions where this occurs. Images were taken at t = 10 minutes (positive control), 20 minutes (UFH only), and 18 minutes (+histones, +protamine). Experiments were performed >4 times. Scale bars represent 20 µm.

We next explored whether the interaction between heparin and histones may be of *functional* relevance. In aPTT clotting assays, addition of exogenous histones (from calf thymus; contains multiple histone subtypes), neutralises the anticoagulant effect of heparin (Fig. 5a). We then assessed the influence of histones on fibrin formation under flow. When recalcified whole blood is perfused over immobilised collagen, fibrin formation occurs after 6.9 ± 0.22 minutes (Fig. 5b). As expected, heparin blocks fibrin formation. However, when heparinised blood is supplemented with histones or protamine, fibrin formation is restored. (Figure 5b; onset of fibrin formation 13.7 ± 1.74 and 15.6 ± 0.94 minutes for protamine and histones, respectively).

**Figure 6:**
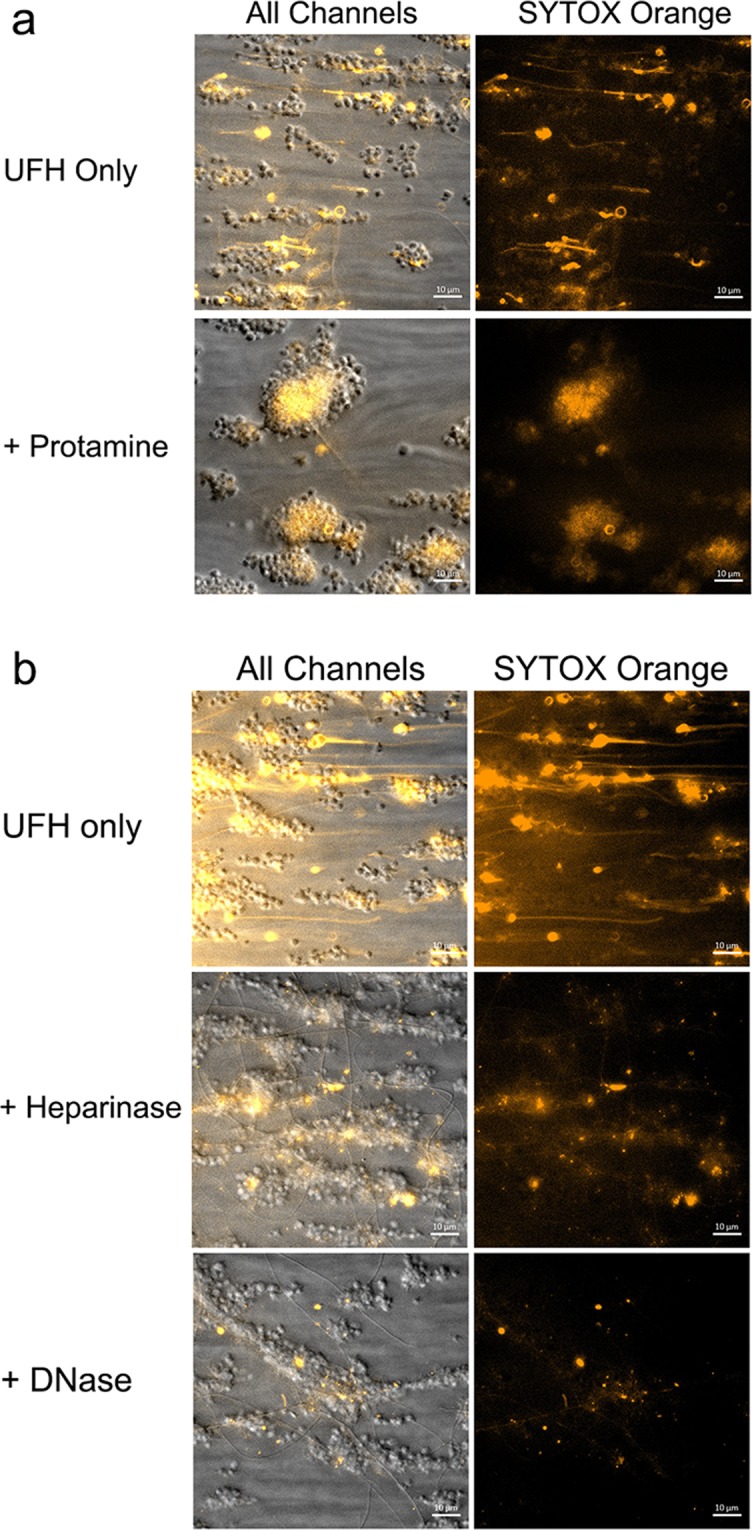
Disruption of polymer formation. (**a**) by preincubation of 10 IU/mL heparin with protamine sulfate (125 µg/mL). (**b**) by preincubation of 10 IU/mL heparin with Heparinase I/III (5 U/mL) or DNAseI (10 µg/mL). Experiments were performed thrice, scale bars represent 10 µm.

### DNase I and heparinase I/III both interfere with SYTOX-positive polymer formation

We so far observed that SYTOX-positive polymers exclusively emerge in the presence of clinically relevant levels of heparin. Surprisingly, we were unable to detect several heparin-binding proteins on these polymers. Instead, we identified histones, which together with SYTOX are features of (extracellular) DNA. Interestingly, SYTOX-positive polymer formation is disrupted in the presence of protamine, suggesting that heparin is directly involved in polymer formation (Fig. 6a). In order to determine the composition of the polymers, we targeted polymers with DNase I or heparinase I/III. We found that both DNase an Heparinase I/III effectively disrupted polymer formation (Fig. 6b; quantification in Fig. S5). In control aPTT clotting assays, we confirmed the ability of the heparinase to reverse the anticoagulant effects of heparin (Fig. S6). Altogether, our findings show that the SYTOX-positive polymers contain DNA and that their formation is dependent on the (anticoagulant) activity of heparin.

## Discussion

In this live-cell imaging study, we made the surprising discovery that heparin triggers the formation of polymers in flowing whole blood that can be visualised with SYTOX. Further characterization of these polymers revealed that their length reversibly increases with increasing shear rate. In addition, they contain histone H1/H3, but not AT, PF4, or fibronectin. The interaction between histones and heparin has been described earlier^9^ and in line with these reports we found that histones neutralise heparin’s anticoagulant activity both in coagulation experiments (aPTT) and under flow (fibrin formation). Finally, polymer formation is disrupted by DNase I, heparinase I/III, and protamine, indicating that the polymers contain cell-free DNA (cfDNA), which possibly forms a ternary complex with heparin.

**Figure 7:**
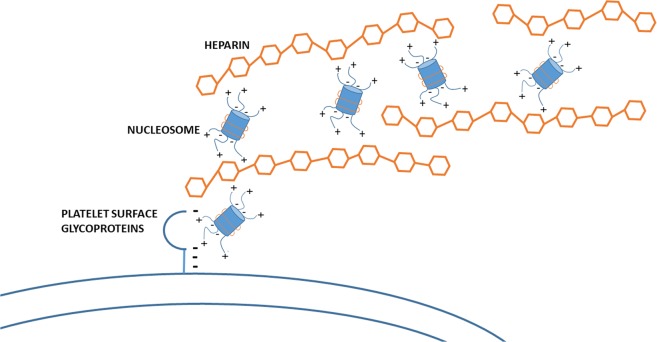
Proposed mechanism for the formation of polymers between heparin and histones/DNA on the surface of platelet aggregates. Negatively charged heparin molecules (in orange) sequester circulating nucleosomes in plasma (in blue) by binding to protruding positively charged N-termini of histones. Under flow, these complexes attach to one or more currently unidentified platelet surface receptors, which we hypothesise to be glycoproteins.

In this study, we have not conclusively shown that heparin directly interacts with SYTOX. In theory, heparin may be able to form polymers through intermediary heparin-binding proteins. In agreement, it was previously suggested that heparin forms a ternary complex with DNA and histones^22^. Furthermore, the heparin-like glycosaminoglycan heparan sulfate – an important element of the glycocalyx – is involved in the processing of circulating chromatin in mice^23^. Another study showed that heparan sulfate binds to nucleosomes through interaction with the cationic tails of histones. The authors suggested that this facilitates polymer formation^24^. We propose we have visualized a similar mechanism in our flow studies (Fig. 7). The fact that no polymers were observed in the presence of pentasaccharide suggests that heparinoid length plays a role, with a heparin molecule having to be able to bind multiple nucleosomes/DNA fragments in order to form these polymers. Nonetheless, the striking similarities of the polymers with DNA are difficult to overlook: they bind SYTOX, contain histones, and show shear-dependent elongation^25–27^.

The effects of heparin on cfDNA have been topic of discussion^28–30^. In addition, several studies suggested that heparin promotes necrosis/apoptosis of leukocytes^31–34^, which may be sufficient to generate the amount of polymers observed in our experiments. However, we consider large scale heparin-induced leukocyte cell death improbable as there is (to our knowledge), no clinical evidence which suggests the extent and speed of the effects observed in our experiments. On the other hand, other studies report that heparin is able to dismantle neutrophil extracellular traps (NETs)^35^, and solubilises DNA^36^. Although the origin of the DNA in our study remains unclear, to our knowledge, this is the first report to visually show interplay between heparin and DNA. Our observations may be of interest to various fields of research, primarily that of NETosis and studies regarding cfDNA (as a biomarker). Indeed, in NETs research, blood collected in heparin tubes – yielding a final heparin concentration of 18 IU/mL – is often used^37^. In addition, the application of heparin in *in vivo* subjects of NETosis studies may present an additional confounder.

The interaction of heparin with histones abrogates both the cytotoxic effects of histones as well as the anticoagulant effect of heparin^9,38,39^. We confirmed the latter in our experiments. In addition, histones are present in the blood of healthy volunteers^21^. At present, it is unclear where these originate from, i.e. whether these are released as a result of venipuncture/blood sampling, *ex vivo* cell death, or are naturally present in the circulation (e.g. from necrosis/apoptosis, minor tissue damage etc.). In addition, it is tempting to hypothesise that heparin triggers toll-like receptor-mediated release of DNA. At any rate, circulating histones and DNA disturb haemostasis in critically ill patients^40–42^. As such, elucidating the interplay between histones, DNA, and heparin and their combined effects on haemostasis may be relevant to our understanding and treatment of coagulopathy in the critically ill.

## Methods

### Materials and reagents

Citrated blood (3.2% trisodium citrate; 9:1 blood-to-citrate ratio) from healthy participants was obtained through the Mini Donor Service, a blood donation facility for research purposes that is approved by the medical ethics committee of the University Medical Center Utrecht and for which all donors have provided written informed consent, in accordance with the declaration of Helsinki. All participants reported being healthy and free from antiplatelet drugs or non-steroid anti-inflammatory drugs for at least 10 days prior to blood donation. Medical grade heparin solutions were obtained through the hospital pharmacy. These included Unfractionated Heparin (UFH) 5,000 IU/mL for intravenous administration (LEO Pharma, Ballerup, Denmark), Low Molecular Weight Heparin (LMWH; dalteparin) 25,000 IU/mL single dose syringe injections (Pfizer, Capelle aan den IJssel, The Netherlands), and fondaparinux (“pentasaccharide”) 5 mg/0.4 mL single dose syringe injections (Aspen Pharma, Dublin, Republic of Ireland). Protamine sulfate from salmon (P4020), histones from calf thymus (H9250), and heparinase I and III blend (H3917) were obtained from Sigma-Aldrich and dissolved in 10 mM HEPES, 145 mM NaCl supplemented with 500 nM D-glucose (HEPES Tyrode Buffer) pH = 7.4 (HT pH = 7.4) to make a 10 mg/mL (for protamine and histones) or 100 U/mL (for heparinase) solution, respectively. DNase I (10104159001) was obtained from Roche and was dissolved in HT pH = 7.4 to make a 10 mg/mL solution. PPACK (a thrombin inhibitor; FPRCK-01) was obtained from Haematologic Technologies Inc and reconstituted to a 50 mM solution in saline. Immunostaining was performed with Alexa-488 conjugated sheep anti-human-antithrombin (Affinity Biologicals, Ancaster, ON, Canada; SAAT-AP). Additional mouse anti-human AT monoclonal antibodies (used at a concentration of 2 µg/mL) were generated in-house. Furthermore, mouse anti-human Histone H1, which is known to crossreact with histone H3 and - to a lesser extent - H2B (Santa Cruz, sc-8030; Clone AE-4; 1 µg/mL (http://www.merckmillipore.com/NL/en/product/Anti-Histone-H1-Antibody-clone-AE-4,MM_NF-05-457), mouse anti-human PF4 (R&D Systems, Minneapolis, MN, USA; MAB7951; 1.5 µg/ml), and mouse anti-Fibronectin (Abcam, Cambridge, United Kingdom; ab6328-250; 2 µg/mL) were used in combination with a goat anti-mouse APC-conjugated secondary antibody (Invitrogen, Bleiswijk, The Netherlands; A865; 4 µg/mL). Isotype control was performed using the Pierce Mouse IgG2a Isotype Control (Thermo Scientific, Rockford, IL, USA; PA5-33236; 0.5 µg/mL) and Mouse IgG1 Isotype Control (Southern Biotech, Uden, The Netherlands; 0102-01 1 µg/mL). Finally, von Willebrand Factor (VWF) was stained with sheep polyclonal anti-VWF-FITC (Abcam; ab8822; 10 µg/mL) or with rabbit polyclonal anti-VWF (DAKO, Denmark; A0082; 15 µg/mL) in conjunction with goat anti-rabbit-APC (R&D Systems; F0111; 1:200); and an in-house generated AF647-conjugated anti-GPIb-α VhH (200 ng/mL) was used to stain the platelet aggregate surface.

### Live cell imaging under flow

Experiments were performed as described elsewhere^19,43^. In short, cover glasses were cleaned overnight in chromic acid and coated for 90 minutes at room temperature (RT) with 1:10 Horm Type I Collagen (Takeda Pharmaceutical Company, Tokio, Japan) in HT pH = 7.4. Subsequently, the glasses were blocked in 1% Human Serum Albumin (HSA; Fraction V; MP Biomedicals, Illkirch, France) in HT pH = 7.4 (from henceforth 1% HSA/HT) either for 60 minutes at RT or overnight at 4 °C. Cover glasses were mounted on a Perspex single-channel laminar-flow chamber and fastened onto a 0.125 mm thick silicone sheet by applying a 10 kPa vacuum to create an air- and watertight circuit, which was then flushed and primed with HT pH = 7.4. Citrated blood with 1.5 µM SYTOX Orange (Invitrogen) either with or without a single heparinoid (10 IU/mL UFH or LMWH; 0.5 µg/mL pentasaccharide) was perfused through this system at a high shear rate (800 s^−1^) for 5 minutes. Where indicated, only the heparinoid was added to the citrated blood. Subsequently, SYTOX in HT buffer (i.e. without heparin) was perfused over preformed aggregates for 3 minutes to assess whether SYTOX itself is involved in or required for polymer formation. Finally, for the three-dimensional imaging, citrated blood was supplemented with 10 IU/mL LMWH and SYTOX Orange and perfused for 5 minutes at 800 s^−1^, after which HT buffer was perfused and images were acquired. All experiments were performed at RT on a Zeiss Axio Z1 Observer with Colibri LEDs, ZEISS Filtersets 10 (FITC), 20 (Rhodamine), and 50 (APC), and ZEN 2 Blue Edition software. Excitation settings were 83% LED intensity for 300 ms at 470 nm or 555 nm for SYTOX Green and Orange respectively, 50% for 200 ms at 470 nm for FITC, 25% for 200 ms at 470 nm for Alexa Fluor 488, and 75% for 200 ms at 625 nm for APC. Recordings were made either at 400x or 1000x magnification with ± 12 frames per minute for 5–15 minutes. After each perfusion, we took snapshots and obtained image stacks under flow at other areas of interest throughout the perfusion channel. Three-dimensional visualisation was performed by applying the deconvolution module (ZEN software 2.6) with constrained iterative algorithm on an image stack of 34 images (0.35 µm slice size, total image height 11.55 µm).

In the shear dependency experiments, a higher dose of UFH was used in order to get more events (i.e. more polymers). As such, perfusions were performed with citrated blood supplemented with 100 IU/mL UFH and 1.5 µM SYTOX Orange. When a sufficient number of polymers was observed, the pump was turned off for approximately 2 minutes before the start of recording. Next, the shear rate was increased every 10 frames (≈50 seconds) up to a maximum of 800 s^−1^, after which the pump was turned off again for 20 frames (≈100 seconds). This cycle was repeated twice per recording. Polymer length was measured at every second-to-last frame per shear rate using the Zeiss ZEN 2 Blue edition software.

For the detection of fibrin formation, an in-house generated anti-fibrin Alexa Fluor 488-conjugated VhH was used. Perfusions were performed as follows: the nanobody (5 µg/mL) was added to citrated blood, after which heparin (10 IU/mL) premixed with either protamine sulfate (125 µg/mL) or histones (500 µg/mL) was added. Citrated whole blood was recalcified directly before the start of the perfusion by adding 10 µL of a 1 M CaCl_2_ solution per mL of blood (10 mM CaCl_2_). Perfusions were continued until evident fibrin formation became visible, or maximally up to 20 minutes.

Digestion of the SYTOX-positive polymers was assessed by supplementing citrated whole blood with 10 IU/mL UFH, 50 µM PPACK, 6.6 mM CaCl_2_, and 3.1 mM MgCl_2_ (both from a 20x stock solution to prevent dilution) and either DNase I (10 µg/mL) or heparinase I/III (5 U/mL). Subsequently, the blood sample was incubated for 40 minutes at 37 °C, after which 1.5 µM SYTOX orange was added and perfusions were performed at 800 s^−1^ shear rate. In other experiments, 10 U/mL UFH was incubated for 40 minutes with protamine (125 μg/mL) in absence of CaCl_2_ or MgCl_2_ prior to perfusion.

### Immunostaining

After the flow experiments, cover glasses were fixed for 10 minutes in 4% paraformaldehyde (PFA) in HT pH = 7.4 at 800 s^−1^ after which the coverslips were carefully removed from the perfusion chambers and fixated with 4% PFA for another 60 minutes at RT. Subsequently, the cover glasses were rinsed thrice with HT pH = 7.4, incubated with 0.15% Glycine in 1% HSA/HT for 15 minutes at RT before blocking with 1% HSA/HT for 60 minutes at RT. Then, the cover glasses were rinsed an additional five times and incubated for 60 minutes at RT with a 1% HSA/HT solution containing fluorescent dyes (i.e. SYTOX Orange/Green) and/or a primary antibody. If incubation with additional (secondary) antibodies was required, the coverslips were washed a further five times with HT pH = 7.4 before incubation with 1% HSA/HT containing the antibody. Thereafter, the glasses were washed thrice with HT pH = 7.4, twice with demi-water and then mounted in 10% (m/v) Mowiol 40–88 and 2.5% (m/v) DABCO in 20% Glycerol and 200 mM TRIS-HCL, pH = 8.0.

### Activated partial thromboplastin time (aPTT)

Plasma from 4 healthy donors was pooled and used for aPTT measurements. All measurements were performed at 37.2 °C using the MC-10 coagulometer (Merlin Medical, Tredegar, United Kingdom). In short, 50 µL of plasma was warmed up in the cuvette for 2 minutes, then 50 µL of undiluted Dade Actin FS (Siemens Healthcare Diagnostics, Marburg, Germany) was added and after another 2 minutes 50 µL of 25 mM CaCl_2_ was added and clotting time was measured.

### Statistical analysis

Data were processed using the GraphPad Prism statistical package, version 7.04 (GraphPad Software, San Diego, CA, USA). Continuous variables are presented as mean with standard deviation. Differences between groups were analyzed by means of a Friedman test with Dunn correction for non-normal distributed continuous repeated measures data. In all tests, a two-tailed p-value < 0.05 was considered statistically significant.

## Supplementary information

Supplemental Figures

Supplemental Video

## Data Availability

All data generated or analysed during this study are included in this published article (and its Supplementary Information Files).

## References

[1] Boneu B, Caranobe C, Sie P (1990). Pharmacokinetics of heparin and low molecular weight heparin. Baillieres. Clin. Haematol..

[2] Olson ST, Björk I, Bock SC (2002). Identification of critical molecular interactions mediating heparin activation of antithrombin: Implications for the design of improved heparin anticoagulants. Trends Cardiovasc. Med..

[3] Wang L, Brown JR, Varki A, Esko JD (2002). Heparin’s anti-inflammatory effects require glucosamine 6-O-sulfation and are mediated by blockade of L- and P-selectins. J. Clin. Invest..

[4] Nelson RM (1993). Heparin oligosaccharides bind L- and P-selectin and inhibit acute inflammation. Blood.

[5] Wildhagen KCAA (2014). Nonanticoagulant heparin prevents histone-mediated cytotoxicity *in vitro* and improves survival in sepsis. Blood.

[6] Wang F (2015). Heparin defends against the toxicity of circulating histones in sepsis. Front. Biosci. Landmark Ed..

[7] Ekaney ML (2014). Impact of plasma histones in human sepsis and their contribution to cellular injury and inflammation. Crit. Care.

[8] Iba T (2015). Heparins attenuated histone-mediated cytotoxicity *in vitro* and improved the survival in a rat model of histone-induced organ dysfunction. Intensive Care Med. Exp..

[9] Longstaff C (2016). Neutralisation of the anti-coagulant effects of heparin by histones in blood plasma and purified systems. Thromb. Haemost..

[10] Wen Z (2016). Circulating histones are major mediators of systemic inflammation and cellular injury in patients with acute liver failure. Cell Death Dis..

[11] Balhorn R (2007). The protamine family of sperm nuclear proteins. Genome Biol..

[12] Lewis JD (2004). Histone H1 and the origin of protamines. Proc. Natl. Acad. Sci. USA.

[13] Ausió J (1999). Histone H1 and evolution of sperm nuclear basic proteins. J. Biol. Chem..

[14] Eirín-López JM, Frehlick LJ, Ausió J (2006). Protamines, in the footsteps of linker histone evolution. J. Biol. Chem..

[15] Krauel K (2018). Fibronectin modulates formation of PF4/heparin complexes and is a potential factor for reducing risk of developing HIT. Blood.

[16] Gupta K, Gupta P, Solovey A, Hebbel RP (1999). Mechanism of interaction of thrombospondin with human endothelium and inhibition of sickle erythrocyte adhesion to human endothelial cells by heparin. Biochim. Biophys. Acta.

[17] Maccarana M, Lindahl U (1993). Mode of interaction between platelet factor 4 and heparin. Glycobiology.

[18] Prechel MM, Walenga JM (2013). Emphasis on the Role of PF4 in the Incidence, Pathophysiology and Treatment of Heparin Induced Thrombocytopenia. Thromb. J..

[19] Verhoef JJF (2017). Polyphosphate nanoparticles on the platelet surface trigger contact system activation. Blood.

[20] Poletti LF (1997). Structural aspects of heparin responsible for interactions with von Willebrand factor. Arterioscler. Thromb. Vasc. Biol..

[21] Garcia-Gimenez JL (2017). A new mass spectrometry-based method for the quantification of histones in plasma from septic shock patients. Sci. Rep..

[22] Longstaff C (2013). Mechanical stability and fibrinolytic resistance of clots containing fibrin, DNA, and histones. J. Biol. Chem..

[23] Du Clos TW (1999). Chromatin clearance in C57Bl/10 mice: interaction with heparan sulphate proteoglycans and receptors on Kupffer cells. Clin. Exp. Immunol..

[24] Watson K, Gooderham NJ, Davies DS, Edwards RJ (1999). Nucleosomes bind to cell surface proteoglycans. J. Biol. Chem..

[25] Hirano K (2018). Stretching of single DNA molecules caused by accelerating flow on a microchip. J. Chem. Phys..

[26] Hur JS, Shaqfeh ESG, Babcock HP, Chu S (2002). Dynamics and configurational fluctuations of single DNA molecules in linear mixed flows. Phys. Rev. E. Stat. Nonlin. Soft Matter Phys..

[27] Jo K, Chen Y-L, de Pablo JJ, Schwartz DC (2009). Elongation and migration of single DNA molecules in microchannels using oscillatory shear flows. Lab Chip.

[28] Dabi Y (2018). Autoimmune disorders but not heparin are associated with cell-free fetal DNA test failure. J. Transl. Med..

[29] Grömminger S (2015). The influence of low molecular weight heparin medication on plasma DNA in pregnant women. Prenat. Diagn..

[30] Burns W (2017). The association between anticoagulation therapy, maternal characteristics, and a failed cfDNA test due to a low fetal fraction. Prenat. Diagn..

[31] Adachi I, Iwaki H, Adachi H, Ueno M, Horikoshi I (1986). Heparin-induced leukocyte lysis *in vitro*. J. Pharmacobiodyn..

[32] Erduran E, Tekelioğlu Y, Gedik Y, Yildiran A (1999). Apoptotic effects of heparin on lymphoblasts, neutrophils, and mononuclear cells: results of a preliminary *in vitro* study. Am. J. Hematol..

[33] Erduran E, Zaman T, Deger O, Tekelioglu Y, Bahadir A (2012). *In vitro* determination of apoptotic effect of heparin on lymphoblasts by using flow cytometric DNA analysis and measurements of caspase-9 activation and cytochrome C level. J. Pediatr. Hematol. Oncol..

[34] Manaster J (1996). Heparin induces apoptosis in human peripheral blood neutrophils. Br. J. Haematol..

[35] Fuchs TA (2010). Extracellular DNA traps promote thrombosis. Proc. Natl. Acad. Sci..

[36] Courvalin JC, Dumontier M, Bornens M (1982). Solubilization of nuclear structures by the polyanion heparin. J. Biol. Chem..

[37] Carmona-Rivera, C. & Kaplan, M. J. Induction and Quantification of NETosis. In Current Protocols in Immunology 115, 14.41.1–14.41.14 (John Wiley & Sons, Inc., 2016). 10.1002/cpim.16 27801512

[38] Elaskalani O, Abdol Razak NB, Metharom P (2018). Neutrophil extracellular traps induce aggregation of washed human platelets independently of extracellular DNA and histones. Cell Commun. Signal..

[39] Noubouossie DF (2017). *In vitro* activation of coagulation by human neutrophil DNA and histone proteins but not neutrophil extracellular traps. Blood.

[40] Alhamdi Y, Abrams ST, Lane S, Wang G, Toh C-H (2016). Histone-Associated Thrombocytopenia in Patients Who Are Critically Ill. JAMA.

[41] Johansson PI, Windeløv NA, Rasmussen LS, Sørensen AM, Ostrowski SR (2013). Blood levels of histone-complexed DNA fragments are associated with coagulopathy, inflammation and endothelial damage early after trauma. J. Emerg. Trauma. Shock.

[42] Gould TJ, Lysov Z, Liaw PC (2015). Extracellular DNA and histones: double-edged swords in immunothrombosis. J. Thromb. Haemost..

[43] Barendrecht, A. D. *et al*. Live-cell Imaging of Platelet Degranulation and Secretion Under Flow. *J*. *Vis*. *Exp*., 10.3791/55658 (2017). 10.3791/55658 PMC5612051 28715386

