# Supplementary material for: Heparin Forms Polymers with Cell-free DNA Which Elongate Under Shear in Flowing Blood
Source: Sci Rep. 2019 Dec 4;9:18316. doi: 10.1038/s41598-019-54818-3 (PMC6892814; doi:10.1038/s41598-019-54818-3)
Supplement: Supplementary file 1 — Supplemental Figures [file 41598_2019_54818_MOESM1_ESM.pdf]

Heparin Forms Polymers with Cell-free DNA Which Elongate Under Shear in Flowing Blood

Joost C. de Vries[1], Arjan D. Barendrecht[1], Chantal C. Clark[1], Rolf T. Urbanus[1], Peter Boross[2], Steven de Maat[1], and Coen Maas[1*]

[1] Department of Clinical Chemistry & Haematology, University Medical Centre Utrecht, Utrecht, The Netherlands
[2] Immunotherapy Laboratory, Laboratory for Translational Immunology, University Medical Centre Utrecht, Utrecht, The Netherlands

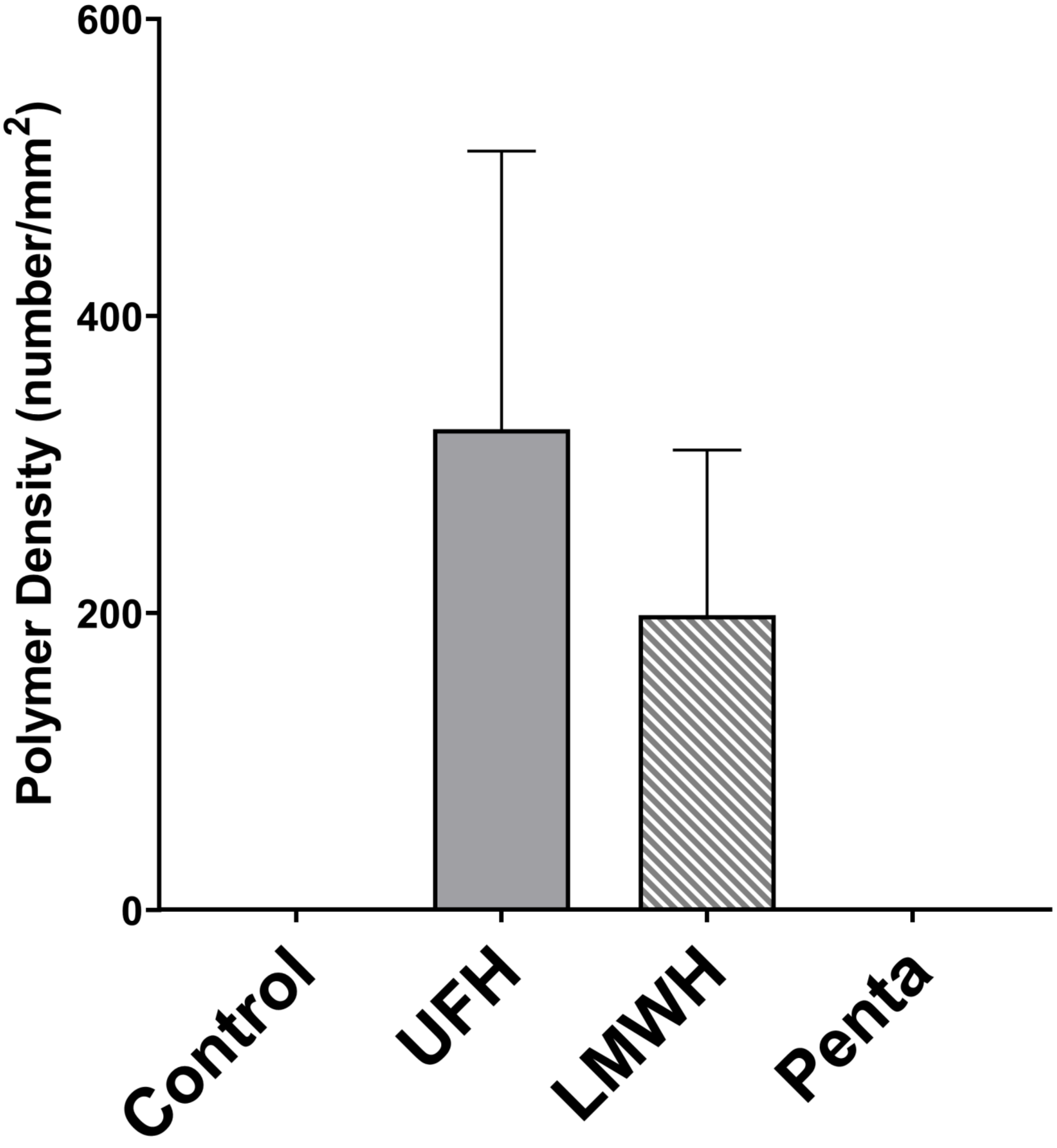

Fig. S1 Quantification of polymer density. The number of polymers per field of view was counted in at least 4 different images per experiment (final frame from recordings, representative slice of an image stack or snapshot which were obtained at random places in the perfusion channel) with analysis of images from three separate experiments per condition. Data are presented as mean ± SD.

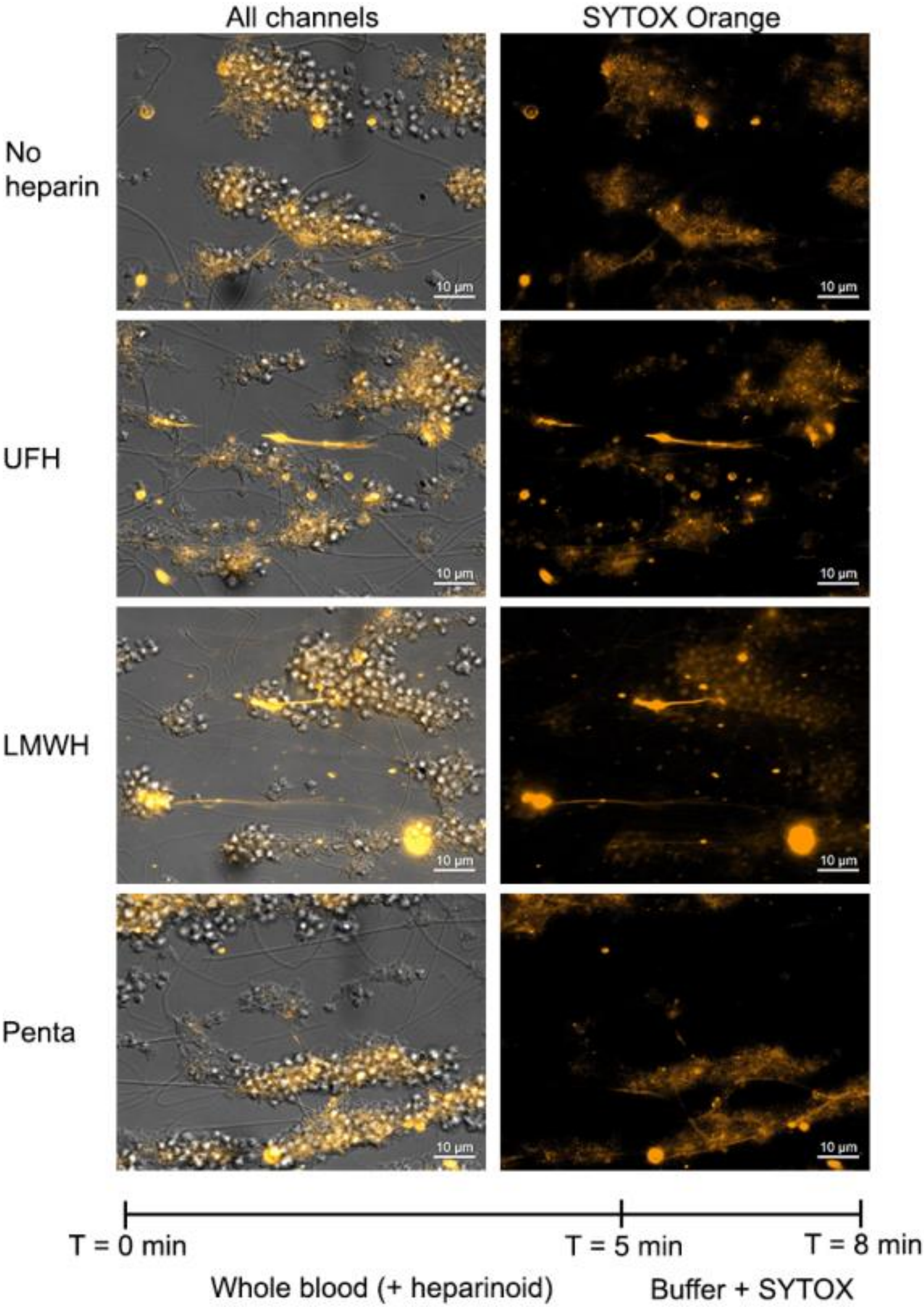

All channels
SYTOX Orange
No heparin
UFH
LMWH
Penta
10 µm
T = 0 min
T = 5 min
T = 8 min
Whole blood (+ heparinoid)
Buffer + SYTOX

Fig. S2. SYTOX is not directly involved in polymer formation. To assess whether SYTOX itself is involved in the polymerisation, platelet aggregate formation in citrated whole blood, supplemented with heparinoids, was first allowed to take place in the absence of SYTOX. SYTOX in buffer was subsequently perfused in a second stage. A timeline of the experimental setup is presented at the bottom of the figure. As before, polymers are visible in the presence of UFH or LWMH, but not in the absence of either, nor in the presence of pentasaccharide. Experiments for all conditions were performed at least thrice. The scale bars represent 20 μm.

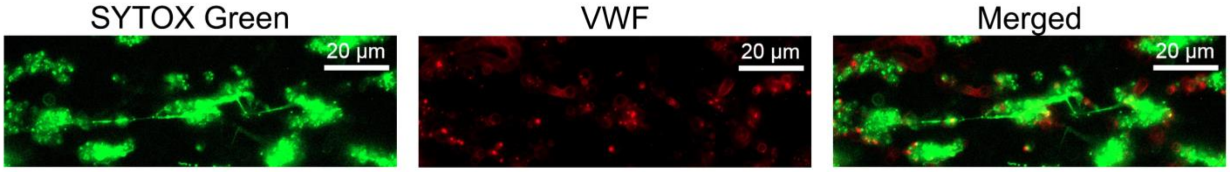

Fig. S3. SYTOX-positive polymers do not contain von Willebrand Factor (VWF). Post-stains were performed in duplicate, scale bars represent 20 μm.

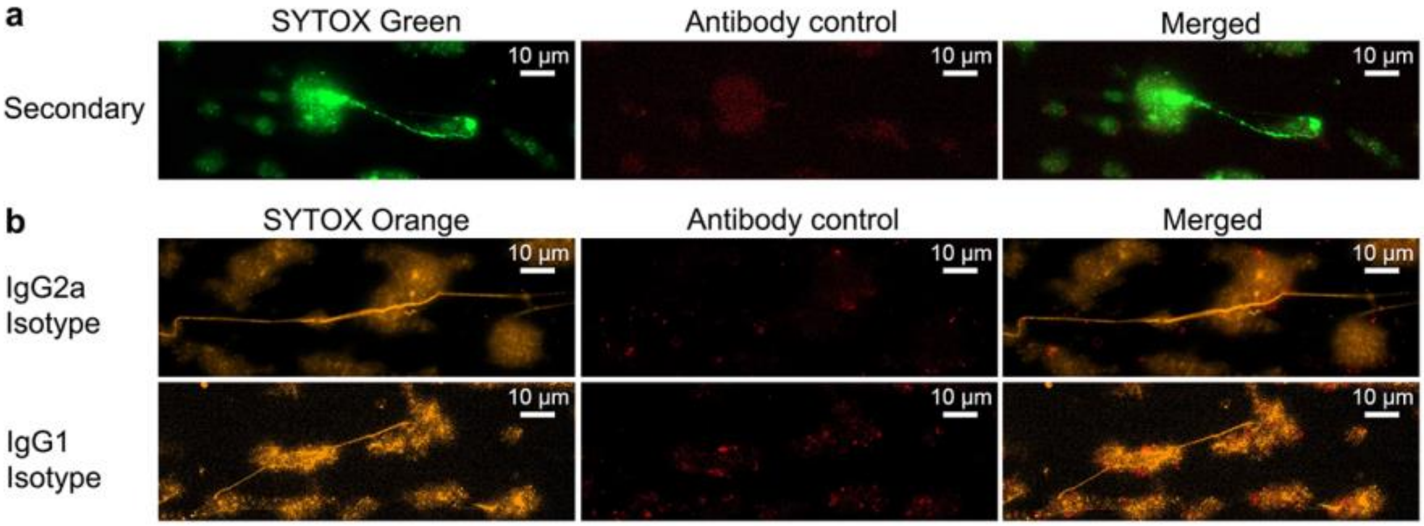

Fig. S4. Antibody controls show no specific signal or colocalisation with the SYTOX-positive polymers. (A) using only the goat anti-mouse secondary antibody – which was used as secondary antibody for all stains in Fig. 4 stains **(B)** isotype controls for the primary antibodies used to stain histones and fibronectin (IgG2a) or antithrombin (IgG1). All experiments were performed at three times, scale bars represent 10 μm.

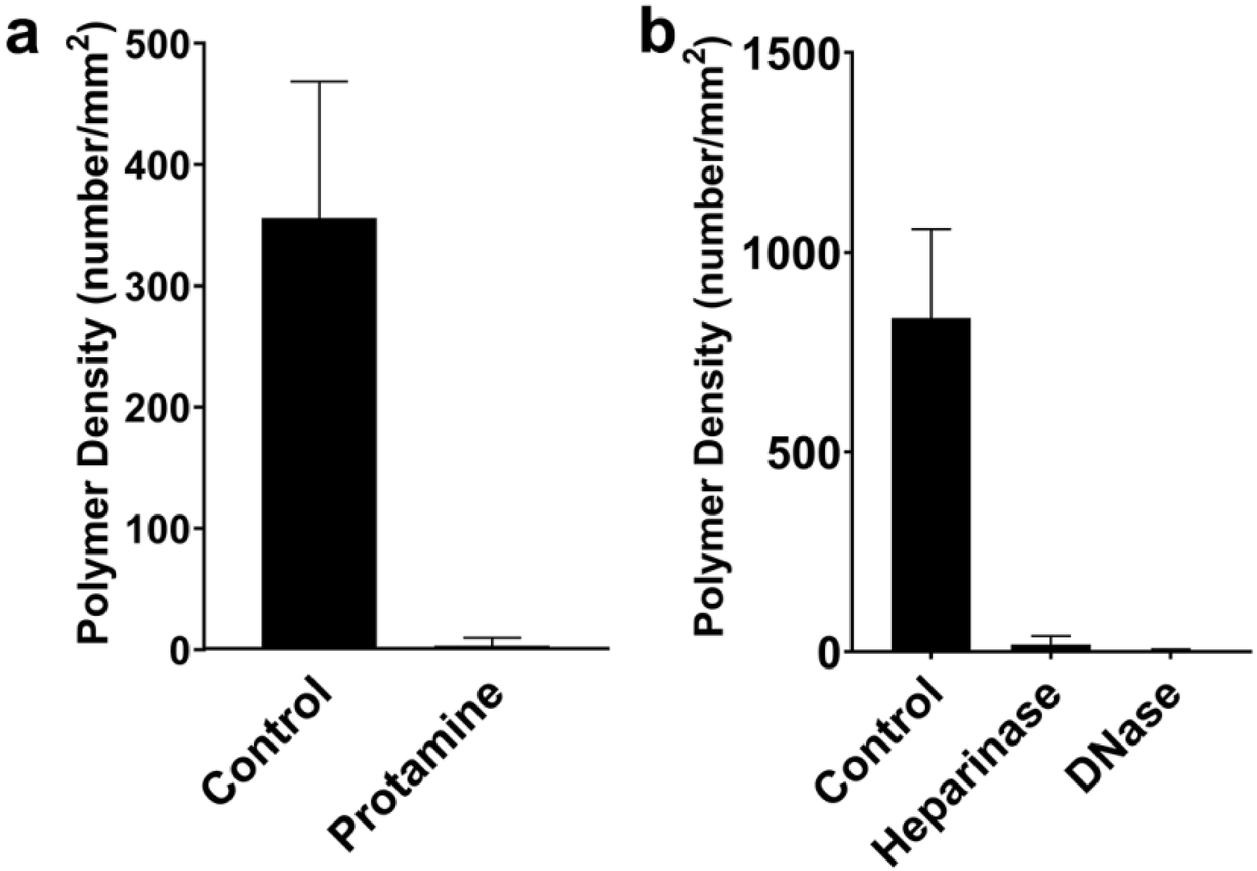

Fig. S5. Quantification of the polymer density. he number of polymers per field of view was counted in at least 4 different images per experiment (final frame from recordings, representative slice of an image stack or snapshot) which were obtained at random places in the perfusion channel) with analysis of images from three separate experiments per condition. Data are presented as mean ± SD. **(A)** Control compared to protamine **(B)** Control compared to heparinase and DNase.

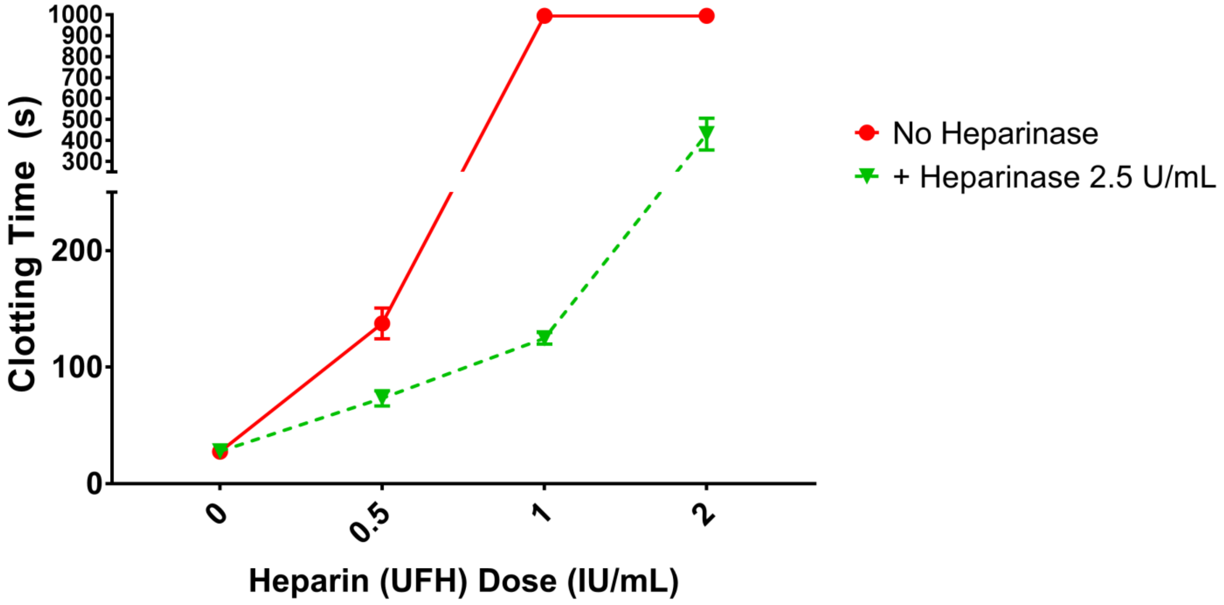

Fig. S6. Heparinase digests unfractionated heparin and normalises aPTT clotting times.

Measurements were performed in triplicate. Data represent means +/- SD.
